# Differential Role of Adipose Tissues in Obesity and Related Metabolic and Vascular Complications

**DOI:** 10.1155/2016/1216783

**Published:** 2016-09-27

**Authors:** Almudena Gómez-Hernández, Nuria Beneit, Sabela Díaz-Castroverde, Óscar Escribano

**Affiliations:** ^1^Biochemistry and Molecular Biology Department, School of Pharmacy, Complutense University of Madrid, Madrid, Spain; ^2^CIBER of Diabetes and Associated Metabolic Diseases, Madrid, Spain; ^3^Instituto de Investigación Sanitaria Hospital Clínico San Carlos, IdISSC, Instituto de Salud Carlos III, Madrid, Spain

## Abstract

This review focuses on the contribution of white, brown, and perivascular adipose tissues to the pathophysiology of obesity and its associated metabolic and vascular complications. Weight gain in obesity generates excess of fat, usually visceral fat, and activates the inflammatory response in the adipocytes and then in other tissues such as liver. Therefore, low systemic inflammation responsible for insulin resistance contributes to atherosclerotic process. Furthermore, an inverse relationship between body mass index and brown adipose tissue activity has been described. For these reasons, in recent years, in order to combat obesity and its related complications, as a complement to conventional treatments, a new insight is focusing on the role of the thermogenic function of brown and perivascular adipose tissues as a promising therapy in humans. These lines of knowledge are focused on the design of new drugs, or other approaches, in order to increase the mass and/or activity of brown adipose tissue or the browning process of beige cells from white adipose tissue. These new treatments may contribute not only to reduce obesity but also to prevent highly prevalent complications such as type 2 diabetes and other vascular alterations, such as hypertension or atherosclerosis.

## 1. Introduction

Obesity is a multifactorial chronic disease with an increased incidence in developed countries over the last decades. Nowadays, it represents a worldwide epidemic [1]; in 2014, 39% of adults older than 18 years showed overweight, and 13% were obese. Obesity is a huge public health problem due to the associated risk with developing other diseases [2]. In this sense, 44% of diabetes cases worldwide, 23% of ischemic heart disease, and 7–41% of certain cancers are attributable to overweight and obesity. This occurs, at least partially, because of the obesity-induced insulin resistance and the fact that adipose tissue is not only an energy reservoir but also a secretory endocrine organ of cytokines, hormones, and proteins that affect the functionality of cells and tissues all over the body [3].

In mammals, the adipose tissue is composed of at least two kinds of adipose tissue, the white adipose tissue (WAT) and the brown adipose tissue (BAT) which have different morphology, distribution, gene expression, and function. WAT is the main energy reservoir and secretes a huge number of hormones and cytokines that regulate metabolism and insulin resistance [3, 4]. The development of obesity depends not only on the balance between food intake and energy expenditure but also on the balance between white adipose tissue, as the main energy reservoir, and brown adipose tissue, specialized in energy expenditure through nonshivering thermogenesis via the mitochondrial uncoupling protein 1 (UCP-1). In addition, BAT could affect body metabolism and alter insulin sensitivity [5, 6] as well as modifying the susceptibility to develop obesity [7]. Moreover, in this review, we also analyze the role of perivascular adipose tissue (PVAT) in obesity and mainly its action in the associated vascular complications. This tissue is located around the arteries and other systemic vessels and depending on the vascular bed may have more or less characteristics of white or brown adipose tissue.

## 2. Differential Morphology, Innervation, and Distribution of Adipose Tissues

### 2.1. WAT

Adipocyte from WAT has a variable shape, although it is classically spherical sized between 25 and 200 *μ*m. In addition, it has a peripheral and flat nucleus with a thin cytoplasm that contains a single large lipid drop, which occupies 90% of the cell volume. It presents few mitochondria and a small smooth and rough endoplasmic reticulum. WAT is composed of adipocytes that are held together by a poorly vascularized and innervated connective tissue [8]. Sympathetic innervation has been described in WAT, although relatively sparse compared to BAT [9]. As occurs in BAT, WAT parasympathetic innervation is controversial and, at the moment, there is a lack of evidence in this regard [10–12]. Finally, the sensory innervation of WAT is histologically known for decades, but its function was revealed more recently; it seems that sensory innervation is essential in the regulation of sympathetic innervation by forming feedback loops [13].

In addition to adipocytes, WAT contains macrophages, leukocytes, fibroblasts, cell progenitors, and endothelial cells. The presence of fibroblasts, macrophages, and other leukocytes, along with adipocytes, realizes the great variety of proteins that are secreted by WAT under varying conditions. White adipose tissue is distributed over the entire body and has different compartments that vary in terms of cell size [14, 15], metabolic activity, and its potential role in insulin resistance and other vascular complications associated with obesity [16, 17].

In humans, two main depots of white adipose tissue are differentiated: subcutaneous depot corresponding to the adipose tissue located under the skin (80% of total fat) and the visceral depot. There are two types of visceral adipose tissue: mesenteric and omental [18]. The first one is wrapped around the intestine; the second one extends from the lower part of the stomach, covering the abdomen, and is normally used in the study of visceral fat. In obesity, ectopic lipid deposition occurs mainly in liver, muscle, and heart. Over years, it is well known that the subcutaneous and visceral adipose tissues have different molecular, cellular, and anatomical features [19, 20]; for example, the irrigation of both tissues is different [21], and the mRNA levels of leptin in the subcutaneous adipose tissue are increased as compared to the visceral adipose tissue [19]. These tissues are also different in terms of the capacity for fatty acid mobilization [22]. Thus, omental fat is more sensitive to the lipolytic effects of catecholamines and less sensitive to the antilipolytic effects of insulin; therefore, this tissue has a greater capacity for fatty acid mobilization and release into the portal circulation than the subcutaneous reservoir [21, 23].

### 2.2. BAT

The brown adipose tissue consists of brown adipocytes and remaining stroma vascular fraction (SVF) including adipocyte cell progenitors [8]. Thus, the brown adipocyte has a polygonal shape with an oval and centered nucleus on a large cytoplasm that contains multiple and small lipid droplets. It has a large number of mitochondria and an underdeveloped endoplasmic reticulum. In addition, BAT is highly vascularized and innervated [8]. While the sympathetic innervation of BAT is evident [24–26], the parasympathetic innervation is controversial and it seems to be confined to the mediastinal [27] and pericardial BAT [28]. In addition to sympathetic and parasympathetic innervation, it has been described that BAT has sensory innervation; however, the information about the role of this innervation is scarce [29–31].

Originally, it was thought that the BAT was only present in humans during the neonatal period. However, more recently, data have shown that adults retain some metabolically active depots of BAT that respond to cold and sympathetic activation of the nervous system [32]. Such depots are UCP-1 positive and are detected by positron emission tomography (PET) [32]. Currently, in humans, brown adipose tissue has been detected in cervical, supraclavicular, paravertebral, mediastinal, para-aortic, and adrenal regions [32]. In addition, small groups of brown adipocytes inside of the skeletal muscle were also found in mice [7]. On the other hand, recent data have shown that brown adipocytes found inside white adipose tissue depots are not derived from myf5 lineage, such as the classic brown adipocytes of the interscapular tissue of rodents, and are known as “beige” or “brite cells” [33–35]. These cells are positive for UCP-1, with high respiratory capacity, with characteristics of both white and brown adipose tissues and being highly responsive to the polypeptidic hormone irisin [36]. In this sense, it has recently been shown that the exercise-induced irisin secreted by skeletal muscle induces the “browning” of subcutaneous white adipose tissue. However, this protein has little effect on the classic brown adipocytes isolated from the interscapular reservoir [37]. These results suggest that the responsiveness to irisin might be a selective feature of beige cells localized inside of subcutaneous white adipose tissue and improve metabolic and vascular complications associated with obesity [37–40]. Besides its implication in thermogenesis, recent studies have shown that brown adipose tissue could be involved in the reduction of triglyceride and glucose levels and also serve as a source of adipokines playing a different role in the inflammatory response as compared to WAT [41–43].

### 2.3. PVAT

Perivascular adipose tissue is located around the coronary artery (or epicardial adipose tissue), the aorta (periaortic adipose tissue), and other systemic vessels as well as the microcirculatory bed of the mesenteric, muscle, kidney, and adipose tissue, with the exception of the brain circulation [44]. It joins the adventitious layer without any laminar structure or organized barrier. Depending on the vascular bed, PVAT may have more or less characteristics of white or brown adipose tissue. Thus, it has been described that PVAT from the abdominal artery would be essentially white adipose tissue; the PVAT in human coronary arteries would have an intermediate phenotype between brown and white adipose tissues and the PVAT from thoracic aortic artery would be very similar to the brown adipose tissue [45, 46]. Functionally, similar to BAT, it has been described that lipid clearance and maintenance of intravascular temperature were impaired in response to cold exposure in mice lacking PVAT [47]. Vascularization and innervation of the PVAT considerably vary with location and this could explain the different functional features of PVAT. Indeed, it has been shown that PVAT of human saphenous vein also receives direct sympathetic innervation [48].

## 3. Adipose Tissues as Endocrine Organs

### 3.1. WAT

The white adipose tissue is not only an energy reservoir but also a secretory organ of certain molecules that have endocrine, paracrine, and autocrine actions [49]. Some of these molecules secreted by adipocytes are involved in the regulation of body weight (leptin, adiponectin), in the local inflammation generated in obesity (TNF-*α*, IL-6, and IL-1*β*), in vascular function (Ang II and PAI-1), or in breeding (estrogens, among others).

Leptin is a hormone mainly secreted by the adipocytes that plays an important role in the regulation of body weight through its central effects on appetite and peripheral effects on the regulation of energy expenditure [50]. The vast majority of obese patients present high concentrations of leptin that are increased depending on the degree of adiposity and hyperinsulinemia, which is referred nowadays as leptin resistance [51]. This hyperleptinemia has been involved in the insulin resistance showed by obese subjects through alterations in insulin receptor phosphorylation [52]. Another hormone secreted by adipocytes that participates controlling food intake is adiponectin. In several studies, hypoadiponectinemia has been observed in patients with obesity, diabetes mellitus, and coronary artery disease [53, 54]. In addition to its antidiabetogenic and antiatherogenic effect, it also has an inverse relationship with other risk factors such as blood pressure, total cholesterol, and low density lipoproteins (LDL) [55, 56]. Cross-sectional population studies show that low adiponectin concentrations or high leptin levels are related to an increase in the metabolic and cardiovascular risk [57–59].

Different cell types including the adipocytes secrete several proinflammatory cytokines. They have paracrine or autocrine actions and participate in the local inflammatory response that occurs in the adipocytes of obese patients. It has been described that the levels of TNF-*α* in the adipocyte are positively correlated with the size of the adipose depots [60]. In addition, the levels of mRNA of TNF-*α* are increased in adipose tissue of several murine models of obesity and diabetes and obese patients, linking such increase with the development of insulin resistance [61, 62]. On the one hand, TNF-*α* activates lipolysis and inhibits the expression of LPL and GLUT-4 as a mechanism addressed to reduce the excessive size of fat depots. However, high levels of TNF-*α* in adipose tissue could account for any of the metabolic alterations associated with obesity such as insulin resistance. Thus, TNF-*α* increases free fatty acid levels reducing insulin sensitivity, and, in the liver, it has an inhibitory effect on insulin action increasing the hepatic glucose production [63]. Thus, the neutralization of TNF-*α* using monoclonal antibodies reduces the glucose levels in the murine diabetic KKAy model [64] and improves the glycemic control in insulin resistant subjects [65]. Similarly, treatment with anti-TNF-*α* antibodies for six weeks reduced the fasting hyperglycemia and glucose intolerance and improved insulin sensitivity in visceral white adipose tissue, mainly in gonadal depot from 52-week-old BATIRKO mice, which shows an increased adiposity associated with a severe brown fat lipoatrophy [66]. In this mouse model, treatment with anti-TNF-*α* antibodies reduced activation of NF-*κ*B in both adipose tissues and the expression of proteins controlled by this transcription factor both in the gonadal white adipose tissue and brown adipose tissue and in the aorta [66]. In addition, vascular insulin resistance and dysfunction were reversed by the treatment with anti-TNF-*α* antibodies [66]. Angiotensin and plasminogen activator inhibitor 1 (PAI-1) are also molecules secreted by adipocytes whose gene expression is increased in obesity [67, 68], showing a deleterious effect on vascular function. Moreover, another component of the renin-angiotensin system, also present in adipocytes, is angiotensin II, which has a positive effect on the differentiation of adipose tissue and regulates adiposity owing to their lipogenic actions [69]. In relation to PAI-1 secretion by adipose tissue, an increased production in visceral fat has been observed as compared to subcutaneous fat. In fact, PAI-1 levels were increased in the central obesity related to its associated vascular alterations [68].

### 3.2. BAT

Brown adipose tissue is also an endocrine organ like WAT and secretes different cytokines, hormones, and other factors such as TNF-*α*, adiponectin, and leptin. However, there are a large number of molecules that are also secreted by BAT. Many of these, including fibroblast growth factor type 21 (FGF21), are required to cold adaptation and adrenergic stimulation [70–72]. In addition, FGF21 can also act directly on brown adipose tissue, regardless of the adrenergic control, opening new pathways to explore mechanisms that control body fat [73]. Other proteins secreted by BAT such as adipsin, FGF2, IGF-1, prostaglandins, and adenosine have autocrine actions.

In addition, BAT secretes other proteins such as IL-6 and neurotrophic factors including BDNF (brain-derived neurotrophic factor) and nerve growth factor (NGF), which could have different roles in BAT as compared to WAT [74, 75]. NGF secretion occurs mainly by brown preadipocyte proliferation, which promotes sympathetic innervation triggering greater norepinephrine stimulation. Other paracrine factors, besides the neurotrophic, synthesized by BAT are vascular endothelial growth factor (VEGF), angiotensinogen, and nitric oxide. The expression of VEGF is increased during the proliferation and differentiation of brown adipocytes, in order to maintain a high level of vascularization. Both noradrenaline and cold exposure induce an increased expression of VEGF in BAT [76]. On the other hand, nitric oxide (NO) produced mainly by endothelial nitric oxide synthase (eNOS) might be responsible for the physiological regulation of blood flow as well as for thermogenesis in BAT, and the authors also suggest that eNOS activity and expression may be controlled by sympathetic nerve activity [77].

Unlike the white adipose tissue that is quickly infiltrated by inflammatory cells in response to high-fat diet-induced obesity, brown adipose tissue does not appear to accumulate such infiltrate of macrophages [78]. This may be due to the larger number of mitochondria of BAT, which allows fatty acid metabolism through *β*-oxidation. However, in WAT, the ability to metabolize lipids would be exceeded, having lipotoxic effects, triggering the inflammatory response and facilitating the infiltration of macrophages and immune cells [79]. In this sense, another group has recently demonstrated that the macrophages from brown adipose tissue do not have the same expression profile of cytokines and chemokines as those from white adipose tissue [79].

### 3.3. PVAT

The PVAT like other adipose depots releases adipocytokines, such as adiponectin, leptin, IL-6, and TNF-*α*. PVAT establishes a communication with the other layers of the vessel wall through the* vasa vasorum*, being different from the actions of the other adipose tissues. Moreover, PVAT activity has direct paracrine action in vascular smooth muscle cells from media layer and endothelial cells from intima layer [80]. Thus, the main endocrine actions of PVAT on vascular cells are the regulation of vessel tone in physiological conditions and vessel remodeling in pathophysiological conditions [80]. In this regard, inflammatory cells in PVAT might be implicated in the recruitment and/or proliferation of adventitial myofibroblasts and finally contribute to vascular remodeling. Therefore, in response to vascular damage or high-fat diet, PVAT produces proinflammatory adipocytokines upregulation and adiponectin downregulation [81, 82]. In addition, the prochemotactic activity of PVAT due to the accumulation of inflammatory cells between the PVAT and the adventitia layer of human atherosclerotic aortas has been described [82]. In contrast to other adipose depots, PVAT cells secrete greater amounts of angiogenic factors. So, hepatocyte growth factor (HGF) is mainly secreted by PVAT cells and induces endothelial cell growth and cytokine release from smooth muscle cells [83].

## 4. Role of Adipose Tissues in Obesity-Induced Inflammation and Its Associated Vascular Complications

### 4.1. WAT

There are numerous differences between visceral and subcutaneous adipose tissues related to adipokine secretion [84]. In this sense, peripheral obesity is characterized by an accumulation of subcutaneous adipose tissue and is more frequent in women. This type of obesity is not associated with an increased risk of related pathologies [85]. However, central or abdominal obesity is more common in men and consists of an accumulation of visceral adipose tissue. This type of obesity has been associated, through epidemiological studies, with a higher risk of diseases such as insulin resistance, type 2 diabetes, and hypertension, greatly increasing cardiovascular risk [86].

**Figure 1:**
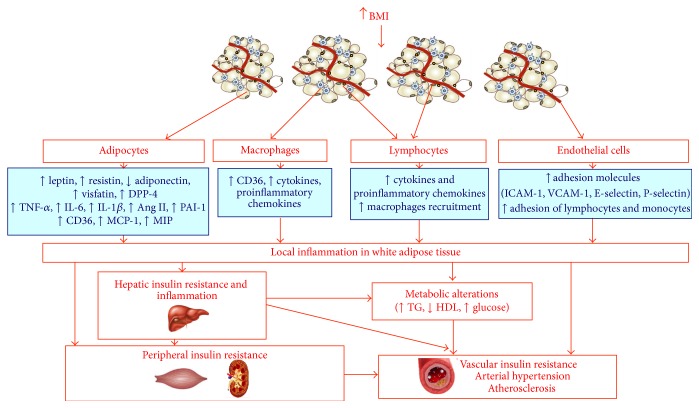
Contribution of white adipose tissue to obesity and its associated metabolic and vascular complications. Obesity is a proinflammatory state of low grade. Adipocytes, infiltrated macrophages, and lymphocytes in addition to endothelial cells from capillaries close to adipocytes contribute to local inflammation in WAT. In obesity an increase of lipid accumulation takes place in adipocytes, triggering cellular stress and the activation of JNK and NF-*κ*B pathways leading to local inflammation in the adipocyte. The inflammation can go through the portal vein to the liver and finally to other peripheral tissues like vascular tissues where it can produce atherosclerosis, hypertension, and vascular insulin resistance.

Under obesity, diet excess and obesity itself produce an accumulation of lipids in adipocytes, triggering cellular stress and the activation of JNK and NF-*κ*B pathways [87, 88]. These inflammatory signaling pathways regulate the phosphorylation of proteins and different transcriptional events that lead to an increase in the production of proinflammatory molecules, including TNF-*α*, IL-6, leptin and resistin, chemokines such as monocyte chemoattractant protein 1 (MCP-1), and other proatherogenic mediators, such as PAI-1. Endothelial adhesion molecules (e.g., ICAM-1 and VCAM-1) and chemoattractant molecules (e.g., CCX) bind to integrins and chemokines receptors (CCR), respectively, and they favor the recruitment of monocytes and other inflammatory cells to the adipose tissue. Internalized monocytes differentiate to macrophages and amplify the inflammatory response producing many of the same inflammatory cytokines and chemokines described above [89] (Figure 1). Some recent articles have also suggested that T cells could play an important role in both the production of proinflammatory cytokines and the recruitment of macrophages to the adipose tissue in obese patients [90]. The lymphocytes infiltration precedes the population of monocytes in response to high-fat diet and could provide proinflammatory mediators, which promote the recruitment and activation of macrophages (Figure 1). Cytotoxic T lymphocytes CD8+ are highly enriched in the adipose tissue of mice subjected to high-fat diet, which is consistent with the significant increase of CD8+ cells in obese patients [90]. Thus, mice deficient in CD8 were partially resistant to develop high-fat diet-induced obesity, while the transfer of CD8+ cells aggravated inflammation of adipose tissue [90].

Besides fat and inflammatory cells, other cell types could participate in the inflammatory response. Thus, the adipose tissue is vascularized with multiple capillaries in contact with each adipocyte [91]. In this sense, for fat expansion, microcirculation could play a key role in adipose tissue inflammation. Thus, leukocytes will not adhere to a normal nonstick endothelium, while endothelium expresses adhesion molecules and binds leukocytes upon high-fat diet administration [92]. Endothelial cells from adipose tissue could increase adhesion proteins, such as ICAM-1, VCAM-1, E-selectin, and P-selectin in response to an increased adiposity and thus promote the adhesion of inflammatory T cells and monocytes [93].

Increased adiposity activates inflammatory response not only in adipocytes but also in the liver through the portal vein [94] (Figure 1). This suggests that lipid accumulation in the liver or steatosis may induce a subacute inflammatory response in this organ, which is similar to the local inflammation observed in adipose tissue that follows lipid accumulation in the adipocyte [94–96]. Proinflammatory molecules produced in abdominal fat through the portal circulation could be responsible for the onset of liver inflammation. In addition, in the fatty hepatocyte, activation of NF-*κ*B and an increase in the expression of cytokines occur, including TNF-*α*, IL-6, and IL-1*β* [94]. Proinflammatory cytokines are involved in the development of insulin resistance and activate the resident hepatic macrophages (Kupffer cells). In obesity, increased adiposity does not increase the number of Kupffer cells but its activation occurs [94]. In the liver, there are different cells types involved in local inflammation and insulin resistance such as immune and endothelial cells [95]. Therefore, the proinflammatory and proatherogenic mediators, which are produced by the adipose tissue and liver and associated with immune cells, generate a systemic inflammation that produces insulin resistance in skeletal muscle and other peripheral tissues. In the vascular tissue, insulin resistance could help to initiate the atherogenic process [96] (Figure 1).

In this sense, it has been described that novel and relevant adipokines as visfatin and dipeptidyl peptidase 4 (DPP-4) are produced by white adipose tissue that might have great impact on cardiovascular complications associated with obesity. So, visfatin had strongly been related to pro-inflammatory factors in severe obesity [97], a novel marker of hypertension in advanced age patients [98] and a predictor of inflammation and endothelial injury in several metabolic diseases [99]. In this regard, it has been demonstrated that visfatin/Nampt might exert direct deleterious actions on the cardiovascular system, including cell proliferation, monocyte/macrophage activation and recruitment, vascular inflammation, and remodeling, all of which leading to the development of atherosclerotic lesions [99]. In addition, DPP4 is also positively correlated with adiposity [100] and insulin resistance in diabetic patients [101, 102]. DPP-4 is a ubiquitous enzyme that regulates incretins and consequently is related to the pathophysiology of Type 2 Diabetes Mellitus. DPP4 is mainly secreted by adipocytes and endothelial cells and acts as a regulatory protease for cytokines, chemokines, and neuropeptides involved in inflammation, immunity, and vascular function [103].

### 4.2. BAT

In mice, the activation of brown adipose tissue reduces adiposity and protects from the high-fat diet-induced obesity [104, 105]. Thus, the loss of BAT mass [5], such as the severe brown lipoatrophy induced by the insulin receptor deletion in that tissue [66, 105], or the loss of UCP-1 [106] confers susceptibility to obesity in mice. In recent years, it has been described that the amount of BAT was inversely correlated with the body mass index in humans, especially in aged people [32]. In addition, it has recently been shown that BAT could protect against multiple diseases associated with ageing [8]. Thus, individuals with smaller depots of BAT are more susceptible to accumulate WAT and to increase body weight showing an increased risk of developing metabolic and vascular alterations [96, 107].

**Figure 2:**
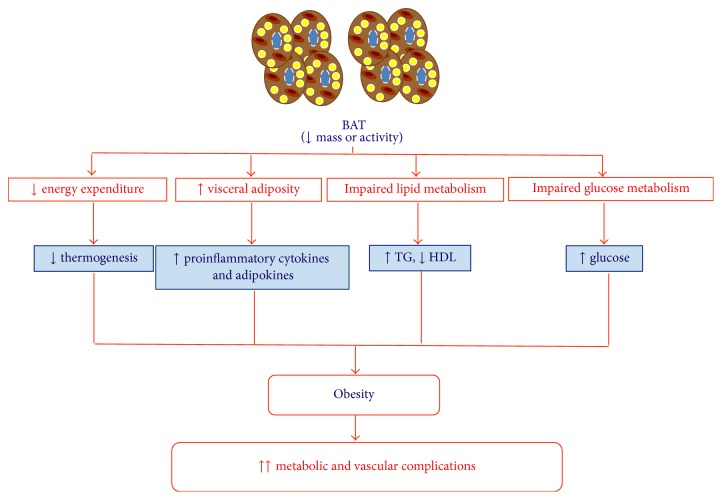
Contribution of brown adipose tissue to obesity and its associated metabolic and vascular complications. One of the possible causes that induce the development of obesity could be a decrease in the amount and activity of the brown adipose tissue. In this situation, there would be an alteration of functions that perform brown adipose tissue on lipid metabolism and carbohydrate as well as the expression profile of cytokines and adipokines, favoring obesity and the related metabolic and vascular complications.

Besides thermogenesis, recent studies have demonstrated that BAT could have a leading role in lipid and carbohydrate metabolism (Figure 2). Firstly, brown adipose tissue may be involved in the reduction of elevated triglyceride concentrations and therefore in the reduction of obesity in humans [41, 108]. Thus, triglyceride-rich lipoproteins (TRLs) carry lipids within circulation, where a portion of fatty acids can be liberated by LPL [109]. Other peripheral organs such as white adipose tissue and skeletal muscle capture fatty acids, while the remnant cholesterol-rich particles are removed by the liver [109]. In addition, high levels of triglycerides and cholesterol-rich remnant particles, as in diabetic dyslipidemia, represent risk factors to develop cardiovascular diseases [110, 111]. It has been described that the increased activity of BAT by short exposures to cold could control the metabolism of the TRLs in mice, by regulating the removal of these lipoproteins and the excess of circulating lipids [41] and thus decreasing the levels of triglycerides and slightly increasing HDL levels (Figure 2). Thus, fatty acids are efficiently introduced into the brown adipose tissue due to a metabolic program that pushes TRLs to a highly efficient uptake of fatty acids. This process associated with an increase in the expression of VEGF [112] leads to an increase of lipoprotein permeability, allowing triglycerides to come out of the capillaries. The BAT switched on by norepinephrine not only activates the fatty acid release from triglycerides and a greater production of VEGF but also increases the expression of LPL [41, 113]. Therefore, LPL degrades triglycerides and allows that fatty acids may be available through plasma membrane transporters as CD36. In addition, it has been shown in humans that activation of BAT by cold exposure increases its oxidative metabolism, reducing triglyceride content and contributing decisively to energy expenditure [114]. Therefore, the activation of BAT would be able to correct the hyperlipidemia, improving the deleterious effects of obesity and dyslipidemia such as insulin resistance or the atherogenic process. So, this year, it has been described that BAT activation reduces plasma triglyceride and cholesterol levels and attenuates diet-induced atherosclerosis development in an experimental model [42]. Initial studies suggest that BAT activation in humans may also reduce triglyceride and cholesterol levels, but potential antiatherogenic effects should be assessed in future studies [42, 114].

On the other hand, it has also been described that BAT could regulate carbohydrate metabolism [41] (Figure 2). The mitochondria from BAT use pyruvate for combustion whenever UCP-1 is activated by fatty acids [115]. In addition, glucose transporters GLUT-1 and GLUT-4, may be involved in the glucose uptake by BAT since the activity and expression of both transporters are augmented by both cold exposure and norepinephrine [41, 116–118].

### 4.3. PVAT

**Figure 3:**
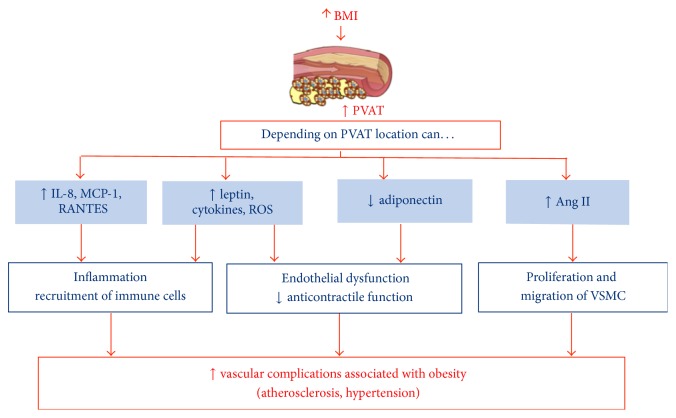
Contribution of perivascular adipose tissue to obesity and its associated metabolic and vascular complications. Perivascular adipose tissue depending on its location interacts with the endothelium, vascular smooth muscle cells, and immune cells. In the same way, there are certain mediators that would be involved in the vascular disorders associated with obesity, such as hypertension and atherogenesis.

Perivascular adipose tissue that extends from adventitious layer is a key modulator of the vascular function in both thin animal models and subjects. However, in pathological conditions especially obesity-related cardiovascular diseases, the beneficial effects of PVAT on vascular functions are impaired (PVAT dysfunction) and transformed into detrimental roles [119]. So, the perivascular tissue increases its size, creating an environment of hypoxia that could decrease the production of adiponectin, which has protective effects against atherogenesis and other vascular complications [46] (Figure 3). Like other adipose tissue depots, PVAT also secretes many biologically active substances that can act in both autocrine and paracrine fashion. PVAT has also a proven role in vascular inflammation [119–121]. On the other hand, it has been described that diet-induced weight loss reverses obesity-induced PVAT dysfunction through a mechanism involving reduced inflammation and increased nitric oxide synthase activity within PVAT [122].

In addition, in obesity and metabolic syndrome, PVAT loses its vasoregulatory capability due to a decreased release of vasodilator adipokines and a simultaneous increase in vasoconstrictor factors release [123]. Thus, the perivascular adipose tissue has anticontractile properties that are lost in obesity [44, 124] (Figure 3). It has also been described that an increased PVAT could be positively correlated with the amount of intra-abdominal adipose tissue [125]. Therefore, in obesity and atherosclerosis, PVAT, in addition to increase its size, can be infiltrated by immune cells, such as macrophages and T lymphocytes [78, 126]. The accumulation of T lymphocytes could favor the expansion of adipose tissue due to adipogenesis stimulation by increased 15d-PGJ2 production and PPAR-*γ* activation [127]. However, macrophages do not affect PVAT expansion but produce cytokines that alter its adipokine secretion [82]. Thus, lower adiponectin levels [124] and elevated leptin levels [81, 127], proinflammatory cytokines and chemokines [128–130], and reactive oxygen species (ROS) [44, 131] and esterified fatty acids [104] have been described in PVAT from both obese patients and animal models of obesity (Figure 3).

However, it has been described that the inflammatory properties of the epicardial adipose tissue are independent of obesity [132]. In this regard, recent studies in mice have also shown that the PVAT surrounding the thoracic aorta artery is very similar to BAT in terms of morphology and gene expression profile [78]. In addition, perivascular adipose tissue in the thoracic aorta together with BAT is more resistant to inflammation induced by high-fat diet [78] (Figure 3). Moreover, the PVAT that has thermogenic properties similar to BAT in rodents and beige fat in humans together with the triglyceride clearance might inhibit the development of atherosclerosis [47]. It would be interesting to check if the perivascular adipose tissue in obese patients with and without cardiovascular disease has a similar morphology and gene expression profile to BAT in the studied murine models. Thus, the activation of BAT phenotype in PVAT could be beneficial in order to prevent vascular diseases associated with obesity, such as hypertension and atherogenesis.

## 5. New Perspectives in the Treatment of Obesity

An early indication for the treatment of obesity along with caloric restriction is physical exercise in dosed way appropriate to the physical condition of each patient. There are considerable evidences that caloric restriction increases the life expectancy [133] and reduces the risk of developing diabetes, cardiovascular disease, degenerative disorders, and some types of cancer [133, 134]. In addition to caloric restriction, there are evidences showing that an energy balance maintained for several months, which includes an increase in energy expenditure, tends to be effective in lowering adiposity. This reduction occurs mainly in visceral fat, which possesses the highest lipolytic activity as compared to adipose tissue from other regions [135]. In addition, people with a good physical condition have greater lipolysis than those inactive [136]. Another aspect that enhances the physical exercise in obese patients is the lipid profile. First, it raises HDL levels and therefore lowers the LDL/HDL ratio and cardiovascular risk [137]. In addition, exercise increases the size of the LDL and HDL particles leading to a less atherogenic lipid profile than those small LDL and HDL particles, typical of obese patients [138]. Moreover, a regular physical exercise also decreases triglyceride levels in those individuals with initially high values, through an improvement in insulin sensitivity [138, 139]. Physical exercise also produces an increase in oxidative potential and thus promotes the metabolism of more lipids and carbohydrates in the aerobic way, producing very desirable peripheral adaptations. Therefore, the physical exercise normalizes metabolic profile and allows the reduction of morbidity and mortality due to these causes [140, 141].

In addition to the role played by peripheral tissues, energy homeostasis is strongly controlled by the Central Nervous System (CNS). Several areas of the brain that constitute cognitive and autonomic brain systems form networks involved in the control of food intake and thermogenesis, also contributing to energy homeostasis [142]. These networks include the dopamine mesolimbic circuit, the opioid, endocannabinoid, and melanocortin systems. The activity of all these pathways is modulated by peripheral factors such as hormones derived from adipose tissue and the gut, which access the brain via the circulation and neuronal signaling pathways to inform the central nervous system about energy balance and nutritional status. The balance between food intake and energy expenditure is achieved via a highly coordinated communication between the executive, reward, and autonomic circuits in the brain and circulating homeostatic signals [143].

Changes in energy stores induced by food deprivation, overfeeding, or excess physical activity lead to adaptations in the controls of energy intake and expenditure that oppose them. These changes are signaled to reward and autonomic SNS circuits by peripheral hormones, such as leptin and ghrelin. Leptin, whose production varies with the size of the adipocytes in WAT [144], can initiate its central actions via the hypothalamus and VTA (ventral tegmental area) [145, 146]. Similarly ghrelin, also influenced by the nutritional status, can also act on the hypothalamus, VTA, and the DVC (dorsal vagal complex) [147, 148]. These regulatory processes seem particularly effective preventing the reduction in energy/fat reserves which seem resolutely “defended.” Such a reduction leads to regulatory responses that promote energy intake [149, 150] and reduce energy expenditure [151], which unpins the difficulty in an individual's ability to combat obesity [152]. The antiobesity agents whose mechanism is based on the control of CNS present a moderate efficacy in the long term [153]; moreover, these agents produce many central compensation and side effects such as headache, dizziness, fatigue, nausea, dry mouth, cough, constipation, paresthesias, taste alterations, insomnia, elevation in heart rate and memory, or cognitive changes [154, 155].

In recent years, several antiobesity drugs designed to limit energy intake have been withdrawn from the market due to serious adverse effects [156]. Nowadays, only two drugs are approved specifically for weight loss by the US FDA: the lipase inhibitor (Orlistat) that is also approved by the European Medicines Agency but has a limited long-term effectiveness [157] and the recently approved novel selective agonist of the serotonin 2C receptor (Lorcaserin) [158]. Thus, more efforts are needed to develop new antiobesity agents. In this regard, strategies designed to increase lipid mobilization and oxidation could be very useful in the treatment of obesity and associated diseases. In this sense, there are some antidiabetic medications in the market that promote weight loss and improve cardiovascular outcomes [159]. So, inhibition of DPP4 enzyme activity increases endogenous intact glucagon-like peptide-1 (GLP-1), thereby stimulating insulin secretion that subsequently lowers blood glucose. Therefore, multiple DPP4 inhibitors have been developed for treating type 2 diabetes [160]. Although various gliptins are known to be neutral on body weight in type 2 diabetic patients, the effect on body fat mass has not been fully elucidated in humans and animals yet [161–163]. However, recently it has been described that fat loss by the DPP4 inhibitor evogliptin, in contrast to exenatide, might likely be mediated by increased energy expenditure and alteration in white adipose tissue metabolism from obese mice [100].

Other pharmacologic treatments for type 2 diabetes are PPAR*γ* agonists as thiazolidinediones (TZDs) because PPAR*γ* regulates multiple pathways involved in the pathogenesis of diabetes, obesity, and atherosclerosis. Previous studies have proposed that these antidiabetic agents might also present diverse pleiotropic effects, such as improvement of the lipid profile [164], endothelial dysfunction [165], and decreased inflammation [166]. In order to avoid side effects associated with TZDs, new drugs have been developed targeting different PPAR isotypes (dual agonists) and more selective PPAR*γ* partial agonists [167, 168]. This year, a new thiazolidinedione, CQ-1777, partial PPAR*γ* agonist improved obesity-associated insulin resistance and dyslipidemia with atheroprotective effects in atherosclerosis mice model. Moreover, CQ-1777 did not affect body weight, food consumption, fat accumulation, or bone density [169].

In order to fight this global epidemic represented by obesity and its associated metabolic and cardiovascular complications, the pharmacological activation of the SNS does not appear to be useful due to negative side effects [170]; scientists must join efforts to advance the knowledge of brown adipose tissue and its promising therapeutic potential against obesity and related complications [169–171]. It has been described that adaptive response of brown adipose tissue to a moderate and intermittent stress through sympathetic activation could increase the proliferation and differentiation of brown adipocyte progenitors and increase mitochondrial mass and UCP-1 expression in this tissue [172]. All of those effects, along with the stimulation of BAT depots in white adipose tissue or skeletal muscle [173–176], could increase energy expenditure and reduce oxidative stress in visceral adiposity. However, some clinical trials performed with *β*3-AR agonists have not achieved a significant response in terms of weight loss and energy balance [177–180]. Interestingly, transplantation of brown adipose tissue (0.1–0.4 g) to the visceral cavity in mice is able to prevent weight gain and improve the glucidic homeostasis in obese mice subjected to high-fat diet [181]. As it has been described, activation of brown adipose tissue deposits in humans, which are composed of beige adipocytes [35], could open a new research line to determine if this type of cells may have some therapeutic potential. In recent years, it has been proposed that fat browning can be used as a therapeutic tool for metabolic disorders and cardiovascular diseases. Firstly, adaptive changes of skeletal muscle in response to exercise include adjustments in the production and secretion of myokines that induce myogenesis and fat browning together with a concomitant increase in energy expenditure [182]. Although exercise has been the most common factor for fat browning [183], there are some other factors implicated. So, browning of WAT can be achieved by several different means [173] including CNS activation modulating sympathetic output to WAT and the recruitment and activation of immune cells. Moreover, WAT browning can be reached by direct action on white adipocytes or beige precursor cells through the activation of PPAR*α* [184], PPAR*γ* [185], FGF21 [186], IL6 [187], natriuretic peptides [188], beta aminoisobutyric acid (BAIBA) [189], gamma aminoisobutyric acid, or JAK inhibition [190]. Recently, two novel factors as musclin and TFAM have been proposed for fat browning [190]. Musclin is a myokine produced by muscle during exercise [191], activates PPAR*γ*, and, therefore, induces WAT browning having beneficial metabolic and cardiac effects [190, 191]. TFAM is a transcription factor involved in mitochondrial biogenesis and, therefore, has also been involved in WAT browning [190].

In addition to musclin, irisin, another novel adipomyokine, is involved in the browning of WAT during exercise in mice models [37]. However, the impact of irisin on white-to-brown transdifferentiation in humans has been heavily questioned [192, 193]. In the last years, it has been proposed that irisin can exert cardioprotector effects [194, 195] and improves endothelial function due to the activation of the AMPK-eNOS signaling pathway [40]. Finally, *α*-lipoic acid promotes mitochondrial biogenesis and brown-like remodeling in cultured white subcutaneous adipocytes from overweight/obese donors [196].

## 6. Conclusions

Finally, given the capacity of brown adipose tissue in energy expenditure and the effects on carbohydrate and lipid metabolism, as well as their potential resistance to inflammation together with perivascular adipose tissue, new perspectives for the treatment of obesity could focus on the design of new drugs or different regimes or therapies that increase the amount and function of brown adipose tissue not only to combat obesity but also to prevent type 2 diabetes and other associated vascular and metabolic disorders.
